# Does a screening digital rectal exam provide actionable clinical utility in patients with an elevated PSA and positive MRI?

**DOI:** 10.1002/bco2.69

**Published:** 2021-05-04

**Authors:** Courtney M. Chang, Andrew G. McIntosh, Daniel D. Shapiro, John W. Davis, John F. Ward, Justin R. Gregg

**Affiliations:** ^1^ McGovern Medical School University of Texas Houston TX USA; ^2^ The University of Texas MD Anderson Cancer Center Houston TX USA

**Keywords:** active surveillance, clinically significant prostate cancer, digital rectal exam, elevated PSA, prostatic magnetic resonance imaging

## Abstract

**Objective:**

To define the value of a digital rectal exam (DRE) in the prostate‐magnetic resonance imaging (MRI) era. Prostate MRI is increasingly used in men with elevated prostate‐specific antigen (PSA) prior to biopsy.

**Methods:**

A retrospective study was performed in men with elevated PSA undergoing MRI followed by MRI fusion with systematic biopsy and men with elevated PSA/active surveillance with negative MRI followed by biopsy. Baseline clinicopathologic characteristics and DRE findings were collected. We examined performance of a positive DRE on sensitivity and specificity of diagnosing clinically significant prostate cancer (CSPC).

**Results:**

A total of 339 patients had elevated PSA and positive MRI followed by MRI fusion guided with systematic biopsy. Pre‐biopsy DRE was documented in 286/339 patients, who were included in further analysis. About 81.6% positive, 78.7% questionable, and 55.8% negative DRE patients had CSPC. Positive DRE had 21.8% sensitivity and 91.3% specificity for CSPC. Positive or questionable DRE had 42.1% sensitivity and 81.5% specificity. Among 148 men with non‐CSPC (GG1)‐targeted biopsy, 28 had systematic biopsy with CSPC. About 5/28 had positive DRE and 8/28 had positive or questionable DRE. Twenty‐seven patients were included who had elevated PSA/on active surveillance with negative MRI and biopsy done within 2 years. About 77.8% had negative, 7.4% had questionable, and 14.8% men had positive DRE. About 7.4% had CSPC and all had a negative DRE.

**Conclusions:**

Our study provides limited evidence for the value of a DRE. However, it does show occasional benefit in detecting GG2 or higher disease and given the lack of cost and side effects, should still be considered.

## 1 INTRODUCTION

The digital rectal exam (DRE) has been a routine part of prostate cancer diagnosis and screening for decades, providing important prognostic information to clinicians. As time has passed, the DRE has remained a mainstay of clinical management and guidelines,^1^, ^2^ even when tools such as prostate‐specific antigen (PSA) testing^3^ and prostate multiparametric magnetic resonance imaging (MP‐MRI),^4^ have been integrated into clinical practice. DRE adds both to risk assessment and stratification following a diagnosis of prostate cancer and is notably efficient in terms of cost (low, although does require an in‐person visit) and ease of performance.^5^, ^6^, ^7^ However, multiple studies have demonstrated variability in both the consistency in performance of a DRE^8^ and its inter‐ and intra‐observer reliability.^9^, ^10^ Given the issues with clinical benefits, some question the utility of subjecting patients to this uncomfortable exam. In addition, other studies have pointed out the DRE exam as a potential barrier to prostate cancer care, especially for underrepresented minorities.^11^, ^12^

Data suggest that prostate MRI may improve the detection of CSPC. The PROMIS study evaluated men with elevated PSA up to 15 ng/mL and no previous biopsy and found MP‐MRI could be used as a triage test to allow men to avoid unnecessary transrectal ultrasound (TRUS) biopsy and diagnose fewer non‐CSPC.^13^ The PRECISION randomized trial demonstrated that among patients with an elevated PSA or abnormal DRE, MP‐MRI‐targeted biopsy improved the diagnosis of CSPC and avoided detection of low‐risk disease compared to standard systematic biopsy.^14^ While the use of MRI‐targeted biopsy alone, without systematic cores, is a current topic of debate,^15^, ^16^, ^17^ its suggestion by these data^15^, ^16^, ^17^ raises the question as to whether a DRE has a role in screening algorithms in which only targeted biopsy of positive MRI lesions is pursued.

Therefore, we sought to define the value of a DRE in the detection of clinically significant (defined as Gleason grade group [GG] 2 or higher) prostate cancer in the setting of pre‐biopsy MRI testing. We aimed to determine the clinical utility of DRE in the setting of an abnormal MRI and explore its use in a subgroup of men with negative MRI.

## 2 METHODS

This is a retrospective study consisting of two cohorts of men. The first cohort consisted of consecutive patients who underwent MRI fusion biopsy at our institution beginning in 2014, who were prospectively entered into an Institutional Review Board approved database. We included men from this database who, due to an elevated PSA (defined by the treating urologist) and a positive MRI, underwent a transrectal prostate biopsy with both targeted ultrasound fusion biopsies and systematic cores. Of note, four men had biopsy following an initial diagnosis of GG1 prostate cancer using systematic transrectal biopsy and subsequent enrollment on active surveillance. Prior to biopsy, a prostate MRI was performed with a 1.5‐Tesla or 3‐Tesla Signa HDx MR scanner (GE Healthcare, Waukesha, WI, USA) or Siemens MR scanner (Siemens, Erlangen, Germany) using an 8‐channel abdominal array coil was used for mpMRI. For the majority of patients, an endorectal coil was used.^18^ MP‐MRI sequences performed included T1‐weighted, T2‐weighted, diffusion‐weighted imaging including calculation of the apparent diffusion coefficient maps, and dynamic contrast enhancement. Two dedicated genitourinary radiologists reviewed and performed MRI interpretation, segmentation, and contouring of region of interests using ProFuse software. Suspicious lesions were graded on a Likert scale from 1 to 5 and any lesion graded ≥ 3 was targeted for biopsy. For targeted, ultrasound fusion biopsies, all biopsies were performed with monitored IV conscious sedation using the Artemis^TM^ system (Eigen, CA). In the same setting, all patients underwent an additional systematic biopsy from sextant regions that did not overlap previously biopsied areas. Patients were excluded if they did not have a DRE result documented.

Baseline clinicopathologic characteristics and DRE findings for this first cohort were then manually extracted from a chart review, which included age, PSA, PSA density, and ethnicity. The DREs obtained from the chart review were either done at the initial encounter or preoperative visit prior to the planned MRI guided fusion and systematic biopsies. DRE was performed by either the attending physician, fellow, resident, or advanced practice practitioner. The DRE was divided into three categories: “negative,” “questionable,” and “positive.” Negative meaning: cT1c, “symmetrical,” “no nodules,” and “benign/smooth.” Questionable meaning: “asymmetry,” “unsure enlargement/engorgement,” “firm findings,” “induration,” and “unable to access due to body habitus.” Positive meaning: anything other than cT1c, “nodules,” “abnormal,” or “concerning.”

Due to interest in evaluating the role of DRE in setting of a negative MRI, we additionally performed an exploratory analysis in a second cohort of patients. These patients were extracted from a retrospective chart review of consecutive patients seen in a MD Anderson institutional active surveillance clinic from 11/2018 to 2/2020, which consisted of patients with an elevated PSA and those undergoing active surveillance for a previously diagnosed very low‐risk prostate cancer. Patients included in this second cohort from this clinic had a negative MRI (defined as PIRADS 1 or 2, Likert scale 1 or 2, “no dominant lesions,” “no definitive tumors,” and “no suspicious lesions”), and subsequent prostate biopsy within 2 years of the MRI. Of note, some patients underwent different biopsy techniques; these included systematic 12 cores using ultrasound guidance alone or transperineal biopsy. Clinicopathologic characteristics and DRE findings were extracted. DRE results were similarly evaluated for sensitivity, specificity, and negative/positive predictive value in relation to CSPC.

Our primary outcome of interest was the performance of a DRE in detecting CSPC, which was defined as GG2 or higher disease, as defined by the 2014 International Society of Urological pathology (ISUP) Consensus Conference.^19^ Dedicated genitourinary pathologists reviewed all slides. We evaluated the sensitivity and specificity of a negative, questionable, or positive DRE for detection of GG2 disease. We calculated descriptive statistics for clinicopathological characteristics of both groups, including median and interquartile range of age, PSA, PSA density, and numbers of targeted and systematic biopsies. ANOVA and Chi‐square test calculations were used to assess baseline differences between groups. All calculations, including determination of sensitivity and specificity, were obtained using Microsoft Excel.

## 3 RESULTS

**FIGURE 1:**
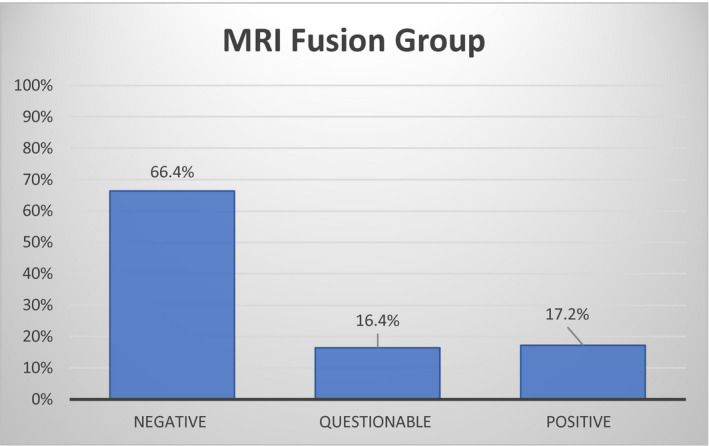
DRE results prior to MRI fusion biopsy. Bar graph represents percentage of patients with DRE classification

A total of 339 patients underwent MRI fusion biopsy with systematic cores due to elevated PSA and positive MRI findings. Pre‐biopsy DRE was documented in 286/339 (84.3%) patients. Therefore, 53/339 (15.7%) did not have a documented DRE and were excluded. Table 1 lists clinical characteristics of the 286 patients included in further analysis. Median age of this group was 66 years (IQR 60‐71) and median PSA was 6.1 (IQR 4.8‐8.9). About 78% were Caucasian, 10% were African American, 4% were Asian, 4% were Latin American, 1% was Middle Eastern, and 3% classified as “other.” The median number of targeted biopsies was 4 (IQR 3‐6) and the median number of systematic biopsies was 10 (IQR 8‐11). DRE results are shown in Figure 1 and are summarized as follows: 49 (17.2%) positive, 47 (16.4%) questionable, and 190 (66.4%) negative.

**TABLE 1:** Clinical characteristics of MRI fusion group (286 patients)

Clinical characteristics of MRI fusion biopsy group				
	Negative DRE (N = 190)	Questionable (N = 47)	Positive (N = 49)	*P*‐value
Age (median, IQR)	66 (60‐70)	67 (61‐71)	67 (61‐71)	.25
PSA (median, IQR)	5.8 (4.5‐8.8)	6.9 (5.6‐13.0)	6.1 (5.1‐9.0)	.04
PSA density (median, IQR)	0.15 (0.09‐0.21)	0.19 (0.11‐0.28)	0.17 (0.12‐0.23)	.03
Race (%)				.57
White	0.67	0.16	0.17	
African American	0.6	0.13	0.27	
Other	0.68	0.21	0.12	
Number targeted biopsy cores (median, IQR)	4 (3‐6)	5 (3‐8)	4 (4‐6)	.24
Number systematic biopsy cores (median, IQR)	10 (8‐11)	9 (6‐11)	9 (8‐11)	.49

Note

*P*‐values calculated using ANOVA or Chi‐Square test when appropriate. IQR = interquartile range.

Table 2 shows the detection of CSPC by DRE results. About 40/49 (81.6%) men with positive DRE and 37/47 (78.7%) men with a questionable DRE had CSPC. Therefore, 77/96 (80.2%) men with questionable/positive DRE had CSPC. About 106/190 (55.8%) men with negative DRE had CSPC. Positive DRE had 21.8% sensitivity and 91.3% specificity for CSPC. Positive DRE had a 81.6% positive predictive value (PPV) and a 39.7% negative predictive value (NPV). Positive or questionable DRE had 42.1% sensitivity and 81.5% specificity. Positive or questionable DRE had a PPV of 80.2% and NPV of 44.2%. Of note, among 148 men with non‐CSPC on targeted biopsy, 28 had systematic biopsy showing CS disease (18.9%). Of these, 5/28 (17.9%) had a positive DRE and 8/28 (28.6%) had a positive or questionable DRE. Table 3 depicts the percentage of CSPC detected based on Likert score and the DRE findings (negative, questionable, and positive) in the first cohort in men with positive MRI. Higher Likert scores corresponded with more CSPC found in all three DRE groups.

**TABLE 2:** Digital rectal exam (DRE) sensitivity and specificity for the detection of clinically significant prostate cancer among men who underwent MRI fusion biopsy

MRI fusion group		
	Sensitivity (%)	Specificity (%)
Negative DRE	57.9	18.4
Questionable DRE	20.2	90.3
Positive DRE	21.8	91.3
Questionable or positive DRE	42.1	81.5

**TABLE 3:** Clinically significant disease detected based on Likert Score and DRE findings

Clinically significant prostate cancer			
N (%)	Negative DRE	Questionable DRE	Positive DRE
Likert 3	15 (14%)	5 (14%)	1 (3%)
Likert 4	28 (26%)	10 (27%)	10 (25%)
Likert 5	63 (60%)	22 (59%)	29 (72%)

Note

About 183 of the 286 patients had clinically significant prostate cancer and were included in this table.

We then evaluated a second cohort of men with elevated PSA or on active surveillance with a negative MRI. Characteristics of this cohort are shown in Table S1. A total of 27 patients with a negative MRI and a prostate biopsy done within 2 years of that MRI were included. Median age was 59 years (IQR 51‐66) and median PSA was 4.8 (IQR 3.5‐6.8). About 56% were Caucasian. About 13/27 (48.1%) of those patients had a GG1 prostate cancer diagnosis and were undergoing active surveillance as treatment, while the others had no prostate cancer diagnosis. About 12/27 (44.4%) of the patients had a transperineal biopsy (TP) with saturation sampling performed; 15/27 (55.6%) had a transrectal ultrasound (TRUS) guided biopsy performed. DRE results are shown in Figure S1 and summarized as follows: 21/27 (77.8%) had a negative DRE, 2/27 (7.4%) had a questionable DRE, and 4/27 (14.8%) had a positive DRE. There were 4/27 (7.4%) patients with CSPC detected and all of those patients had a negative DRE.

## 4 DISCUSSION

To our knowledge, this is the first study to evaluate the performance of DRE in a group selected for fusion biopsy with systematic cores based on elevated PSA and MRI positivity. Our study has shown that a positive DRE had 21.8% sensitivity and 91.3% specificity for CSPC in men with an abnormal MRI undergoing fusion biopsy. When considering a positive or questionable DRE, sensitivity and specificity were 42.1% and 81.5%, respectively. Among 148 men with non‐CSPC (negative or GG1‐targeted biopsy results), 28 had systematic biopsy showing CS disease (18.9%). Of these, 5/28 had a positive DRE and 8/28 had a positive or questionable DRE, suggesting that DRE may add predictive value for men with an abnormal MRI in whom targeted biopsy, only, is planned. Interestingly, in our exploratory cohort of men with negative MRI, there were 4/27 (7.4%) patients who had CSPC detected; however, none of these had a positive or questionable DRE.

Historically, DRE was the fundamental component of prostate cancer screening regimens prior to the advent of the PSA. There have been widely quoted values of the sensitivity and specificity of a DRE in the detection of prostate cancer in the pre‐MRI setting. DRE sensitivity ranges from 69% to 89% and specificity from 84% to 98%. Consistent with these values, positive predictive values range from 25% to 35%.^20^ The DRE is known to be more sensitive for detecting cancer in the peripheral zone rather than in the transition and central zones. While a grossly abnormal DRE can be an indication to go straight to prostate biopsy, a negative DRE provides little reassurance about any absence of intracapsular or extracapsular tumor.^21^ However, the widespread use of the PSA has had a profound impact on prostate cancer screening. A study done by Halpern et al investigated the effects of PSA on the association between DRE and CSPC. Their primary outcome was detection of CSPC diagnosed among men with a suspicious DRE. They found the prognostic utility of a DRE was greater with a higher PSA (PSA > 3), and conversely, was marginal when less than 3. This supported the use of the DRE in men presenting with higher PSA due to improvements in detection of CSPC.^22^

In addition to the PSA test, numerous other tools have been developed to aid in the detection of prostate cancer, replacing the DRE as the sole screening tool. The development of the transrectal ultrasound (TRUS) enabled the detection of lesions that could be biopsied in a more targeted fashion. Shapiro et al sought the clinical utility of a DRE in relation to TRUS. They found TRUS helped to increase the sensitivity of DRE if they both were positive. When TRUS and DRE were positive in 172 cases, a higher cancer yield was obtained with 57.5% of those biopsies being positive. However, when there was a discrepancy in TRUS and DRE, the detection of cancer on a biopsy was low. When TRUS was positive and DRE was negative, the biopsy detected cancer in 13.5% of 172 patients When TRUS was negative and DRE was positive, detection of cancer on biopsies was 10%.^23^ In addition to TRUS, prostate MRI has made a large impact in terms of assisting with risk stratification, clinical staging, and aids in treatment decision‐making in patients with prostate cancer. Comet‐Batlle et al compared the traditional workup using DRE and PSA with DRE, PSA, and endorectal MRI in patients who had elevated PSA and/or abnormal DRE in detection of prostate cancer. They found the endorectal MRI, PSA, DRE combination that had accuracy of 83% in diagnosing prostate cancer, whereas only a DRE and PSA was at 70%.^24^ These studies have shown even with the advent of additional screening tools, DRE has continued to play a role in prostate cancer screening and diagnosis.

MP‐MRI used as a triage prior to prostate biopsy can potentially limit the harms of complications and overdiagnosis faced by men with an elevated PSA. Donato et al reported on a cohort of men undergoing MP‐MRI for suspicion of prostate cancer, followed by systematic TP biopsy for PIRADS 2 lesions and TP‐targeted biopsy with and without systematic cores for PIRADS 3‐5 lesions. They were able to avoid biopsy in 47% of their patients.^25^ The PROMIS study evaluated patients with elevated PSA who underwent MP‐MRI followed by a TRUS biopsy and template prostate mapping (TPM) biopsy (used as reference test), with CSPC defined as ≥Gl 4 + 3 or maximum cancer core length ≥6 mm. They found using MP‐MRI as a triage allowed 27% of patients to avoid primary biopsy and 5% fewer non‐CSPCs were diagnosed.^13^ Furthermore, the PRECISION trial compared patients undergoing MRI with or without a targeted biopsy to men undergoing standard transrectal ultrasound biopsy, defining CSPC as any single core of ≥Gl 3 + 4. They found men who underwent MRI with or without biopsy led to fewer men undergoing biopsy with more CSPC identified and less clinically insignificant prostate cancer detected.^14^ While DRE results are not clear in this study, based on our data, some CSPC may be missed if this approach is utilized without a DRE. Our findings are consistent with studies demonstrating the poor sensitivity of DRE in the pre‐MRI era,^20^, ^26^ though our finding that 8/28 patients with GG2 or higher prostate cancer on systematic cores in whom targeted cores showed only GG1 cancer or benign disease suggest that DRE may have particular relevance in scenarios in which targeted only biopsy is planned based on MRI results.

In a study by Itatani et al, the negative predictive value (NPV) of MP‐MRI was evaluated in 193 patients with negative MRI who then underwent TRUS guided biopsy. Patients were defined as truly negative if after 5 year follow‐up, they continued to have negative findings by DRE, MRI, and repeat biopsy with no increase in PSA. They found the NPV of the MP‐MRI to be 89.6% for CSPC.^27^ Other studies have quoted the NPV of MP‐MRI greater than 95%,^28^ which brings into question its potential use in not only screening of prostate cancer, but also in treatment strategies used with active surveillance. Over the last few years, there has been great interest and shift in the active surveillance paradigms from biopsy‐based surveillance to biomarker and imaging‐based surveillance. Serial MP‐MRIs can provide information in regard to tumor size and characteristics, including vascularity and cell density. Since MP‐MRI typically shows more clinically significant tumors, they can better identify and monitor low‐risk lesions that could progress to intermediate or high‐grade lesions.^29^ The NPV of a stable MP‐MRI in active surveillance candidates was shown to be about 80%, which could help increase the intervals between surveillance biopsies.^28^ While DRE is also included in these active surveillance regimens, it is important to consider our results: among patients with a negative MRI and CS disease, none had a positive DRE, suggesting that other diagnostic tools may be helpful in detecting actionable disease.

Prostate‐specific membrane antigen positron‐emission tomography (PSMA‐PET) has been used increasingly in prostate cancer detection, especially among men with biochemical recurrence after treatment of localized disease. Some studies suggest PSMA‐PET can improve upon tumor localization rates in comparison to mpMRI,^30^ however, no clear role has been delineated. It remains to be seen if PSMA‐PET can definitively improve upon tumor localization in comparison to mpMRI and if DRE could be used to limit the false negatives in the presence of this next‐generation imaging.^31^

Our study is notably limited by sample size, including the fact that not all patients had a DRE. Our results are also confounded by the second cohort of patients undergoing different types of biopsy techniques, including both TRUS and TP with saturation sampling, with TP with saturation sampling tending to be more accurate. Furthermore, DRE positivity was assessed based on a retrospective chart review, introducing selection bias. The performance and outcome of the DRE could have been influenced by known results of the MRI, resulting in omission of a DRE or increased positive DRE rate in a positive MRI. While this methodology awaits validation, it likely differs among performing urologists and would be improved by prospective DRE assessment based on standardized criteria and exam. Finally, a limited number of men were included who had a prior negative MRI and subsequent biopsy, limiting the interpretability of these exploratory results. This fact, coupled with group heterogeneity, limits conclusions that can be derived by this analysis. With the increasing adoption of prostate MRI and incorporation into risk‐assessment algorithms, future studies with increased power will have the ability to better determine DRE performance in the current era.

## CONFLICT OF INTEREST

Intuitive consultant/speaker.

## Supporting information

Supplementary Material
