# Supplementary material for: Does a screening digital rectal exam provide actionable clinical utility in patients with an elevated PSA and positive MRI?
Source: BJUI Compass. 2021 May 4;2(3):188–93. doi: 10.1002/bco2.69 (PMC8988521; doi:10.1002/bco2.69)
Supplement: Supplementary file 1 — Supplementary Material [file BCO2-2-188-s001.docx]

**Supplementary Table 1: Clinical characteristics of Negative MRI Group (27 patients)**

| **Clinical Characteristics of Negative MRI Group** | | | | |
| --- | --- | --- | --- | --- |
|  | **Negative DRE (N = 21)** | **Questionable (N = 2)** | **Positive (N = 4)** | **P value** |
| Age (median, IQR) | 61 (55-67) | 61 (56-65) | 53 (47-58) | 0.23 |
| PSA (median, IQR) | 4.8 (3.5-6.8) | 10.5 (7.2-13.5) | 4 (2.9-5.0) | 0.04 |
| PSA Density (median, IQR) | 0.10 (0.07-0.19) | 0.27 (0.18-0.35) | 0.09 (0.08-0.13) | 0.30 |
| Race (%) |  |  |  |  |
| White | 0.87 | 0.13 | 0 |  |
| African American | 0.86 | 0 | 0.14 |  |
| Other | 0.4 | 0 | 0.6 |  |

P-values calculated using ANOVA or Chi Square test when appropriate. IQR = interquartile range.

**Supplementary Figure 1: DRE results of Negative MRI Group**

Supplementary Figure 1: DRE results of negative MRI group. Bar graph represents percentage of patients with DRE classification.

**Supplementary Table 2: Digital rectal exam (DRE) sensitivity and specificity for detection of clinically significant prostate cancer among men with negative MRI**

| **Negative MRI group** | | |
| --- | --- | --- |
|  | **Sensitivity (%)** | **Specificity (%)** |
| Negative DRE | 100 | 26.1 |
| Questionable DRE | n/a | 91.3 |
| Positive DRE | n/a | 82.6 |
| Questionable or Positive DRE | n/a | 73.9 |
